# Consumer-Led Adaptation of the EsSense Profile^®^ for Herbal Infusions

**DOI:** 10.3390/foods10030684

**Published:** 2021-03-23

**Authors:** Célia Rocha, Ana Pinto Moura, Diana Pereira, Rui Costa Lima, Luís Miguel Cunha

**Affiliations:** 1 GreenUPorto, DGAOT, Faculty of Sciences, University of Porto, Campus de Vairão, Rua da Agrária, 747, 4485-646 Vila do Conde, Portugal; crocha@fc.up.pt (C.R.); dianaspereira25@gmail.com (D.P.); 2 SenseTest, Lda., Rua Zeferino Costa, 341, 4400-345 Vila Nova de Gaia, Portugal; rcl@sensetest.pt; 3 GreenUPorto, DCeT, Universidade Aberta, Rua do Amial, 752, 4200-055 Porto, Portugal; apmoura@uab.pt

**Keywords:** EsSense Profile^®^ adaptation, loose-leaf herbal infusions, harvesting method, focus-groups, lemon verbena

## Abstract

This work aimed to adapt the EsSense Profile^®^ emotions list to the discrimination of herbal infusions, aiming to evaluate the effect of harvesting conditions on the emotional profile. A panel of 100 consumers evaluated eight organic infusions: lemon verbena, peppermint, lemon thyme, lemongrass, chamomile, lemon balm, globe amaranth and tutsan, using a check-all-that-apply (CATA) ballot with the original EsSense Profile^®^. A set of criteria was applied to get a discriminant list. First, the terms with low discriminant power and with a frequency mention below 35% were removed. Two focus groups were also performed to evaluate the applicability of the questionnaire. The content analysis of focus groups suggests the removal of the terms *good* and *pleasant*, recognized as sensory attributes. Six additional terms were removed, considered to be too similar to other existing emotion terms. Changes in the questionnaire, resulting in a list of 24 emotion terms for the evaluation of selected herbal infusions, were able to discriminate beyond overall liking. When comparing finer differences between plants harvested under different conditions, differences were identified for lemon verbena infusions, yielding the mechanical cut of plant tips as the one leading to a more appealing evoked emotions profile.

## 1. Introduction

Although the term “tea” (*chá* in Portuguese) refers to infusions made from leaves of *Camellia sinensis* (L.) Kuntze, in Portuguese colloquial language, it also refers to the wide variety of infusions prepared from dried aromatic plants or parts of plants, such as roots, root-stocks, shoots, leaves, flowers, barks, fruits or seeds other than the leaves of *C. sinensis*. The popularity of these herbal and fruit beverages prepared as infusions reflects the increasing consumer appreciation for the wide range of natural and refreshing tastes and other sensory properties they offer [1].

This reinforces their general social and/or recreational value [2], and their beneficial health properties. They are rich in polyphenols and other functional constituents that possess relatively high antioxidant activities [3]. In addition to water and tea, herbal infusion beverages help to complete proper hydration which is essential for the maintenance of the corporal water equilibrium as well as being part of the Mediterranean diet [4]. They also contribute to a well-balanced diet as they contain no sugar and have almost no calories [5].

Moreover, the marketing of herbal infusions has become increasingly sophisticated and diverse. In Europe, more than 400 different plant parts are used as single or blended ingredients for the preparation of herbal and fruit infusions. Since 2010, the volume of herbal infusion sales has grown in most European countries, by almost 17% [6]. In terms of total volume, Germany is the largest market by far, with sales of herbal infusions amounting to 39,455 tonnes in 2016, contrasting with 19,220 tonnes of tea sold. Even in the United Kingdom, with a traditionally strong tea culture, there has been a shift in sales from black tea to herbal infusions; however, it is still the largest European tea market, representing 107,233 tonnes of tea sold compared to the 4127 tonnes of herbal infusions traded [6].

Facing these extremely competitive markets, this industry requires new strategies to ensure that the production of herbal infusions meets the consumers’ demands. In this context, identification and characterisation of food-elicited emotions have emerged as a competitive advantage in the marketplace for food product differentiation and development. Recent studies indicate that emotional profiles evoked by food products discriminate products more effectively than hedonic measurements [7,8,9]. Going beyond liking may improve the differentiation of similar products in the market. This level of differentiation, beyond liking, may have an impact on better perceiving how consumers react to repeated exposure to different products and may affect the success of new products in the market.

In this context, different emotion lexicons/questionnaires have been developed to study the emotional response elicited by food products, with the assumption that emotions can be easily differentiated by verbal reports—words instead of inference from instrumental measures of participants’ psychological or biological behaviours [10]. For this, Jiang, King and Prinyawiwatkul [9] pointed out that researchers, in their studies on food-elicited emotions methods, should take into account the searching and pre-screening of the emotion lexicon. The first stage should include the identification of terms in the scientific literature, because existing standardized questionnaires represent a strong first step for the construction of the emotion lexicon. The second stage should comprise consumer feedback in the emotion lexicon developed during the first stage, through focus groups or consumer interviews.

According to Jervis and Drake [11], the focus group is a qualitative research methodology in which a moderator guides the interaction between participants to discuss their personal and emotional responses regarding a product or concept. It can be applied in the definition of the product category drivers, in new product concept or product development and to guide the process in consumer needs and expectations [12]. This methodology has been largely used in consumer science for different purposes such as the development of sensory or emotion lexicons, the definition of attributes for other methodologies like conjoint analysis, check-all-that-apply (CATA) or sensory profiling [9,13,14].

In this process, it is important to guarantee that: (i) the suggestions made by consumers correspond to emotion terms; (ii) the meaning of the words is clear for consumers; (iii) there are no redundant words. Subsequently, the researchers should do a pilot study (stage three) to define the final list of emotion terms. The final list should comprise the terms that: (i) have high usage frequency; (ii) were statistically discriminant; and (iii) are appropriate for the consumer [9].

In the last years, various product emotion lexicons and questionnaires have been developed to evaluate foods and beverages in general [15,16,17], specific foods and beverages [8,13,18,19,20], everyday occasions [21], or to evaluate specific sensory dimensions of products, such as the odour of fragranced products [22].

In this context, King and Meiselman [16] developed a method to measure food-evoked emotions for commercial research with different product category users. This questionnaire, known as the EsSense Profile^®^, consists of a list of 39 emotions generated from literature and studies previously published about emotions, together with online surveys and investigations performed at the place of consumption. For the construction of the questionnaire, different emotions were selected: positive (*n* = 19), negative (*n* = 4) and contextual emotions (*n* = 16), contrasting with previous emotion questionnaires, mainly developed in a clinical setting with a particular focus on negative emotions. The approach is to develop a method that is adjusted to the food domain, considering that consumers essentially use positive emotions when describing food and their experiences with food [23]. Moreover, the EsSense Profile^®^ incorporates emotion measures (yes/no or a 5-point intensity scale) combined with the assessment of overall acceptability (9-point scale) to differentiate emotional responses among and within product categories [7,16,24]. Although the EsSense Profile^®^ has been validated using different food categories for its discriminating power [7,8,25], there has been some concern with the length of this questionnaire. The process of asking consumers to rate a long list of 39 emotions on a Likert scale could induce consumer fatigue and boredom and may distort the original consumer emotional reaction [24,26,27]. Additionally, the authors advised that the list of 39 emotions should not be static and widespread to all product categories. Depending on the product specificity, different product categories may be associated with different emotions [7,16]. According to the literature, for a particular food category, pre-defined emotions questionnaires should be complemented with other terms from consumers’ product-evoked emotions, identified when they are thinking about or experiencing the food product [8,9,16,28]. Alternatively, terms may be removed from the previously defined questionnaires, as consumers do not use them when describing their emotional reactions regarding a specific food product [8,16].

Thereby, a shorter version of the EsSense Profile^®^ has been developed, the EsSense25, reducing the original instrument from 39 to 25 terms, using a sorting task to remove words with similar meaning, to make the list faster and easier for consumer testing [29]. This adaptation of the EsSense Profile^®^ showed similar results compared to the application of the original questionnaire, reinforcing the applicability of the EsSense 25 list. Nevertheless, the authors pointed out that the occurrence of context-based perceptual and response biases may affect the meaning of the words. Words can take different meanings depending on the product category, the number of list terms and the translation of emotion terms [30,31]. The EsSense Profile^®^ is recommended when a full characterisation of the emotional response to a food product category is needed, as a longer emotion list will capture subtle variations among consumer heterogeneity in emotional responses [29,30,32]. By contrast, the EsSense 25 questionnaire is designed for high-volume testing in a short time [29].

The format of response through CATA is a natural method for consumers to use, since respondents simply need to select attributes that are relevant to them without having to rate all attributes on a scale [33], thus minimizing time and effort for the respondents. This approach increased the focus on each of the terms presented, reducing the number of emotions selected on a questionnaire, and produced the strongest emotions [9,24,25]. Research by Ng, Chaya and Hort [8] has demonstrated the effectiveness of a consumer-defined emotion lexicon approach to evaluate consumers’ emotional responses using CATA, also reinforced by research by Jaeger et al. [34], clearly showing that the EsSense Profile^®^ can be implemented in the CATA variant without major concerns.

The aim of this research was focused on the methodological approach for consumer-led adaptation of the EsSense Profile^®^ questionnaire [16] for the evaluation of herbal infusions. Additionally, a relevant application of this adaption in a real setting is presented, where the impact of the harvesting conditions, namely the type of cut (manual or mechanical) and the part of the plant (tips and 2nd half leaves), on the intrinsic characteristics of selected herbal infusions was studied.

## 2. Materials and Methods

### 2.1. Experimental Design

To achieve the proposed research goals, two different phases were carried out, with a three-month difference between them, following the Jiang, King and Prinyawiwatkul [9] approach for the emotion lexicon process. In the first phase, the Portuguese version [35] of the 39-item EsSense Profile^®^ was used as a starting point for the definition of the emotion term list, aiming for a complete herbal infusions description. Consumers were asked to evaluate samples of eight different herbal infusions using a CATA ballot with the full EsSense Profile^®^. Different types of loose-leaf herbal infusions were evaluated to achieve a representative sample of this product category, with a wide range of sensory properties: a vast array of colours, aromas and tastes. After this, two focus groups were performed to assess the applicability of the questionnaire to discriminate herbal infusions with different harvesting conditions. In the second phase, to assess the impact of harvesting conditions (the type of cut and part of the plant) on the emotional profile, a large panel of consumers evaluated four types of infusions using the CATA ballot with the adapted list of emotions resulting from the first experiment.

### 2.2. Experiment I: Pre-Selection of Terms from the EsSense Profile^®^

#### 2.2.1. Samples

The samples used in this research were provided by a Portuguese producer of aromatic plants. The production of this farmer is solely based on organic farming, certified in 2005 by Ecocert Portugal. The samples were dried according to their regular commercial procedure (dried in a professional dryer, with an optimal temperature of 35–45 °C, for 72 h) and stored in hermetic bags before being processed and analysed. All infusions were prepared using 4.5 g of whole dried leaves picked from plants and infused in 1.5 L of natural mineral water (Continente, Portugal), following the procedure developed by Cardoso [36].

Eight different commercially available loose-leaf herbal infusions were tested: lemon verbena (*Aloysia triphylla*), lemongrass (*Cymbopogon citratus*), lemon thyme (*Thymus x citriodorus*), peppermint (*Mentha x piperita*), lemon balm (*Melissa officinalis*), chamomile (*Chamomilla recutita*), globe amaranth (*Gomphrena globose*), and tutsan (*Hypericum androsaemum*).

For the preparation of infusions, different steeping times and temperatures were used (Table 1), following two criteria: (i) commercial samples of lemon balm, chamomile, globe amaranth, and tutsan were prepared following producer instructions; (ii) samples of lemon verbena, lemongrass, lemon thyme, and peppermint were prepared following the results of previous work, in which steeping time and temperature were optimized for the consumers’ maximum liking [37,38].

When selected times were reached, leaves were removed by picking the strainer from the teapot. The resulting infusions were left to cool down to 65 °C and then placed in thermally insulated flasks until serving. All samples were served in white porcelain teacups (approximately 100 mL) coded with three-digit random codes, according to the infused plant, in individual booths, under normal white lighting.

Tasters were provided with a porcelain spittoon, a glass of bottled natural water and unsalted crackers (Continente, Portugal), for palate rinse between samples. To guarantee a full appreciation of the herbal infusions, participants were allowed to add castor sugar cubes (2.5 g) and instructed to use the same amount for all samples under evaluation, according to their regular consumption habits.

#### 2.2.2. Sensory Panel

A non-trained panel of 100 naïve tasters (60% female; 40% male; 35.4 ± 11.1 years old) were recruited for the EsSense Profile^®^ validation, all of them being regular consumers of herbal infusions (at least once a week). Panellists were recruited from Sense Test’s consumer database, selected from among residents from the Oporto metropolitan area, North of Portugal, and received a small financial compensation for their participation. The company Sense Test ensures the protection and confidentiality of data through the National Data Protection Commission and accomplished internal conduct. Moreover, informed consent was obtained, and participants were free to quit the evaluation at any time.

#### 2.2.3. Ballot and Questionnaire Format

Paper ballots included two questions: overall liking and elicited emotions evaluation. For each of the eight infusions, consumers were asked to score their overall liking, using a 9-point hedonic scale, ranging from 1—“dislike extremely” to 9—“like extremely”, before rating their emotional responses using the 39-terms original EsSense Profile^®^ lexicon, as a measure to avoid potential hedonic score bias [24]. Emotion terms were selected immediately after the scoring of liking, to evaluate the short-term emotions responses [30]. A CATA response format was chosen in a “Yes/No” forced question format with the intent to induce a higher attention level while keeping the advantages of a faster and simpler task [39]. Emotion terms were presented in alphabetic order for one half of the panel and in the inverse order for the other half of the panel, according to previous authors [8,16], to avoid an effect of the list presentation order.

All samples were presented monadically, in one test session and following a balanced order of presentation, according to a Latin square design, to counterbalance carryover effects [40].

#### 2.2.4. Focus Groups

Consumers from the initial panel were recruited for the focus groups. Considering that the researchers wanted at least 15% participants of the initial panel, two focus groups were performed, with the attempt to have no more than eight participants per group: focus group 1, *n* = 8 (75% female; 25% male; 36.9 ± 5.4 years old); and focus group 2, *n* = 7 (86% Female, 14% Male, 33.6 ± 4.5 years old) [41]. Both focus group discussions took place at the Sense Test’s focus group room, in the Portuguese language, and had a duration of approximately 60 min. Both were conducted by the same moderator, the first author, to ensure consistency in interviewing style [42]. The moderator was assisted by other co-authors in dealing with video recording. After an initial icebreaker introduction, participants were invited to taste all the eight herbal infusions and to mark on a tabular ballot (with all the 39 emotions of the EsSense Profile^®^ questionnaire on the rows, and all the eight herbal infusions on the columns) all the emotions that were evoked by the different herbal infusions. Then, they were also invited to consider and pay closer attention to the emotion terms list from the EsSense Profile^®^. Subsequently, the focus group discussion began, and the moderator guided the discussion considering the most relevant topics to the purpose of this research: (i) the capacity of the terms to describe herbal infusions; ii) the presence of redundant terms; (iii) the evaluation of terms that should be grouped or removed [13,28].

The focus group sessions were video-recorded for accuracy of transcription and analysis, following participants’ informed consent, and the recordings were anonymously transcribed verbatim.

#### 2.2.5. Data Analysis

Simple descriptive statistics of overall liking data were performed and two non-parametric tests, the Friedman and Wilcoxon [43] tests, were applied. All statistical tests were applied at a 95% confidence level (*p* < 0.05).

For each emotional term, a Cochran’s test was applied to identify its ability to discriminate the samples.

The focus group transcriptions were analysed and themes were developed by the researchers, based upon the core themes of the focus group guide, considering similarities and differences of participants’ responses [44]. To illustrate the analysis, direct quotes by the participants were transcribed, serving as a description of the topic explored. The quotes used in this text were translated into English.

A new list of terms for the emotional profile of the eight infusions was determined crossing focus group analysis and the results generated by the previous task (CATA).

Data analysis was performed using the XL-STAT 2020^®^ [45] and IBM SPSS for Windows—version 26^®^ [46] software.

### 2.3. Experiment II: Evaluation of the Impact of Harvesting Conditions on the Elicited Emotional Profile

#### 2.3.1. Samples

The impact of the harvesting conditions on the elicited emotional profile associated with four different infusions was evaluated: lemon verbena (*Aloysia triphylla*), lemongrass (*Cymbopogon citratus*), lemon thyme (*Thymus x citriodorus*) and peppermint (*Mentha x piperita*). Leaves from the different plants were collected from the same farm as the samples used in experiment I. However, these were harvested following a 2^k^ factorial plan according to the type of cut (manual and mechanical) and part of the plant (tips and 2nd half leaves, referred to as 2nd half). All plants came from the same plantation lot and were harvested between spring and summer months, totalling four different batches, for each plant.

All the samples were prepared following the steeping time and temperatures described in Table 1.

#### 2.3.2. Sensory Evaluation

Considering the established previous ballot, 300 naïve tasters (61% female; 39% male; 37.4 ± 12.4 years old) evaluated one of the four types of infusions. The option to have the naïve tasters tasting only samples from one plant aims to avoid the systematic interaction of tasters and emotions presented in the list, minimizing the use of the list in a rational way. These participants were selected based on their regular consumption of loose-leaf herbal infusions and were recruited from the same database as for Experiment I. Consumers were invited to score their overall liking for each sample, and then their emotional profile, following the new emotion list questionnaire previously built to evaluate infusions. The procedure for the sample presentation and the format of the CATA ballot was the same as described in Section 2.2.3.

#### 2.3.3. Data Analysis

To analyse CATA questions, initially, a Chi-square test was used to identify significant differences perceived by consumers between samples for each of the terms [47]. After checking the statistical relation between emotions and samples, the frequency of use of each term was determined, by counting the number of consumers who have used each attribute to describe the samples.

To obtain a two-dimensional representation of the samples, a correspondence analysis (CA) was applied from the previously determined contingency table. This analysis provides a sensory map of the samples, allowing the determination of similarities and differences between samples as well as the features that characterize their attributes [48]. A multidimensional alignment (MDA) was applied to assess the degree of association between products and attributes on the perceptual map [49].

## 3. Results

### 3.1. Pre-Selection of Terms from the EsSense Profile^®^

Table 2 shows the mean results of overall liking for the eight samples evaluated. It is possible to observe a high level of liking for all the samples. The least liked were tutsan, chamomile and globe amaranth infusions.

**Figure 1:**
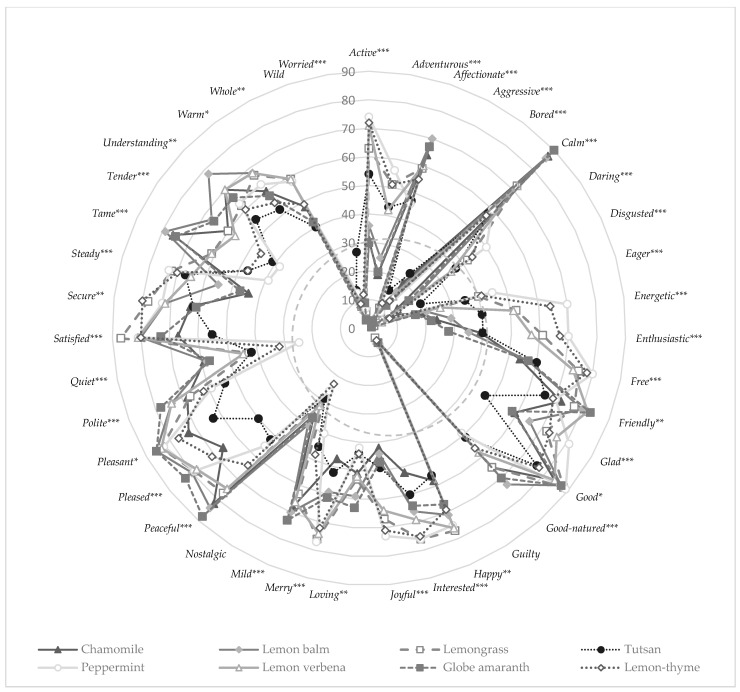
Absolute frequency of elicitation of the different emotion-related terms in the EsSense Profile^®^ questionnaire (*n* = 100). The dotted grey circle represents the 35% citation threshold. Terms yielding significant differences between herbal infusions are marked according to Cochran’s test: *** *p* < 0.001, ** *p* < 0.01, * *p* < 0.05.

The EsSense Profile^®^ consumer test results (*n* = 100) revealed that consumers did not differentiate samples regarding the following emotion terms: *nostalgic* (*p* = 0.102, average citation of 32%), *wild* (*p* = 0.085, average citation of 9%) and *guilty* (*p* = 0.978, average citation of 5%) (Figure 1). These terms were removed because they did not contribute to the differentiation of herbal infusions. Different authors discussed the removal of terms only if quoted by less than 50% of the participants [20] rather than removing terms with less than 20% citation in a checklist questionnaire [16]. In this study, using a forced-choice (yes/no) CATA ballot, authors have decided to apply an intermediate value (minimum citation values of 35%); therefore, the terms *aggressive* (citation range 3–15%), *bored* (citation range 7–24%), *disgusted* (citation range 1–20%) and *worried*, (citation range 6–27%) although discriminating between samples, were removed.

Additionally, according to the focus groups’ content analysis, different emotional terms were suggested for removal from the original list. This was done because consumers considered the terms *good* (*bem*) and *pleasant* (*agradável*) as sensory/hedonic attributes (e.g., “*I interpret that the pleasant is whether the infusion is pleasant or not, and pleased means that if I feel pleased or not with the infusion*”, G2P3).

A few emotion terms were considered as very similar to other emotion terms presented in the EsSense Profile^®^ questionnaire, and therefore removed [9], to obtain a simpler ballot (retained term signalled in **bold**): *happy* (*feliz*) and ***glad*** (*contente*) (e.g., “*I think that glad and happy is also very similar*”, G2P5); *steady* (*firme*) and ***secure*** (*seguro*) (e.g., “*…and for example secure and steady I think the two terms turn out to be the same thing*”, G1P1); *mild* (*meigo*), *tame* (*dócil*) and ***tender*** (*terno*) (e.g., “*are very similar, and make the list very long*”, G1P8 and G1P4); *warm* (*caloroso*) and ***affectionate*** (*carinhoso*) (e.g., “…and I would do the same with the affectionate and warm”, G1P8); *whole* (*completo*) and ***satisfied*** (*satisfeito*), (e.g., “For example satisfied with complete because I think they are very identical”, G1P4).

Using all the previous information, the authors have compiled an emotion list with 24 terms for the discrimination of loose-leaf herbal infusions (see Table 3).

### 3.2. Evaluation of the Impact of Harvesting Conditions on Emotional Profile

#### 3.2.1. Herbal Infusions Comparison

Table 4 shows the mean values of overall liking for each infusion and each treatment (plant part × type of cut) and the aggregate liking for each infusion. From the results, one can observe that all samples have an average value of overall liking higher than 7, with no significant differences between the type of cut (manual and mechanical) and the plant part (tips and 2nd half). From the comparison of the aggregate data for each herbal infusion, one can see that the average values are close to each other.

**Figure 2:**
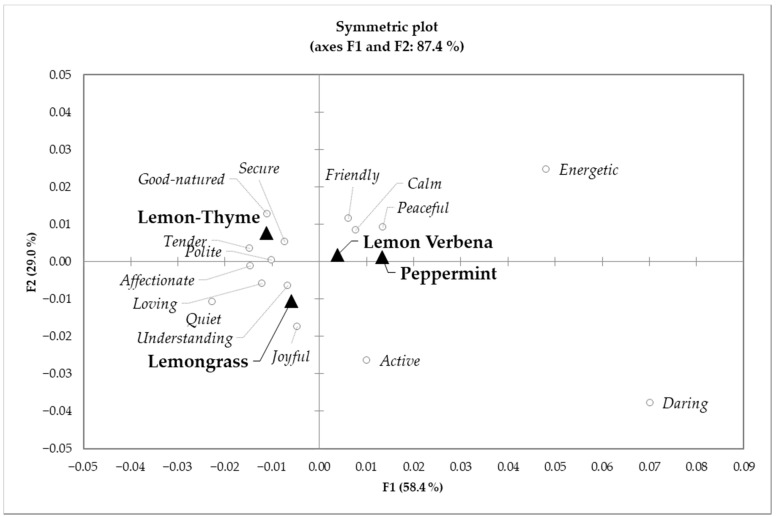
Configurations of samples (triangles) and significant emotion-related terms (circles) in the first and second dimensions of the correspondence analysis applied to check-all-that-apply (CATA) counts from the four infusions: lemongrass, lemon thyme, lemon verbena and peppermint.

On the emotional profile analyses, the authors start with an overview, analysing first the aggregate data (differences between herbal infusions) and then the individual treatment data. Figure 2 shows the configurations of samples and elicited emotion terms in the first and second dimensions of the correspondence analysis applied to the CATA counts for the four herbal infusions: lemongrass, lemon thyme, lemon verbena and peppermint. This configuration explains 90.7% of the total variance of the experimental data. From the analysis of Figure 2, one can perceive that different types of herbal infusions evoke different emotions. Lemongrass samples evoked the following emotions: *joyful*, *quiet*, *active*, *loving* and *understanding*; these emotions are related to affection, liveliness and understanding, and also strongly evoked *daring*, which is related to energy and adventure. Lemon thyme samples evoked the resulting emotions: *polite*, *affectionate*, *secure*, *tender*, and *good-natured* related to security, affection and respect. Lemon verbena and peppermint samples evoked the emotions *calm*, *peaceful*, *friendly*, emotions related to peace, friendship and relaxed, Peppermint also evoked the emotions: *daring*, and *energetic* which are related to happiness, satisfaction, energy and adventure.

**Figure 3:**
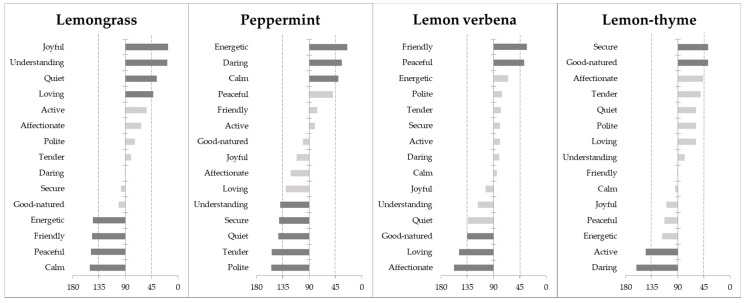
Angles (in degrees) between the vectors from the projections of the four herbal infusions and food-elicited emotion terms in the correspondence analysis map, resulting from multidimensional alignment (MDA) analysis. Dark bars represent the angles below 45°—representing emotion terms positively correlated with the samples, as well as the angles above 135°—representing emotion terms negatively correlated with the samples.

Figure 3 shows the results from the cosines of the angles of herbal infusions with the significant food-elicited emotion terms, resulting from MDA analysis [49]. From this analysis, one may depict in a more detailed way the differences between samples, namely, through their correlation with the different elicited emotion terms.

From Figure 3, one can conclude that the four samples differ in the evoked emotions. The lemongrass infusions are strongly related to *understanding*, *joyful*, *quiet* and *loving* emotion terms and negatively related to *energetic*, *friendly*, *peaceful* and *calm*. For both the peppermint and the lemon verbena herbal infusions, one may observe further insights into the elicited emotions. The peppermint infusions are strongly positively related to *energetic*, *daring* and *calm* and negatively related to *understanding*, *secure*, *quiet*, *tender* and *polite*. While the lemon verbena infusions were merely positively related to *friendly* and *peaceful* emotion terms and negatively related to *good-natured*, *loving* and *affectionate*. The lemon-thyme infusions were strongly related to *secure* and *good-natured* emotion terms, and negatively related to *active* and *daring.*

**Figure 4:**
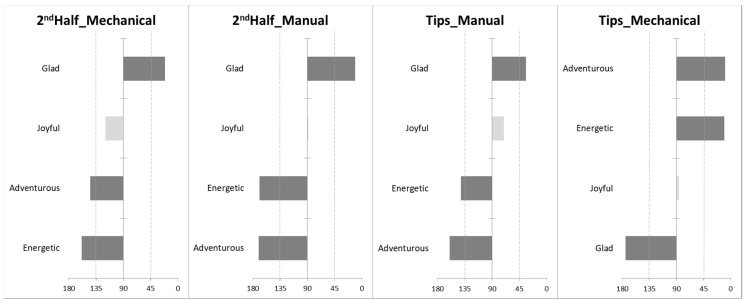
Angles (in degrees) between the vectors from the projections of the lemon verbena infusions (plant part and type of cut) and food-elicited emotion terms in the correspondence analysis map, resulting from multidimensional alignment (MDA) analysis. Dark bars represent the angles below 45°—with emotion terms positively correlated with the samples, as well as the angles above 135°—with emotion terms negatively correlated with the samples.

A closer look into the impact of the type of cut and plant part of each herbal infusion on the elicited emotional profile illustrates differences between treatments of the lemon verbena infusion. Figure 4 shows the only four emotion terms that yield significant differences when describing the emotion-related profile of the samples: *glad*, *joyful*, *adventurous* and *energetic*. The 2nd Half and Tips—Manually harvested products were strongly related to the *glad* emotion and negatively related with *adventurous* and *energetic*, while for Tips from the mechanical harvest the opposite occurs. For the remaining herbal infusions (lemongrass, peppermint and lemon-thyme) no significant differences on the elicited emotional profile between treatments, within each herbal infusion, were found.

#### 3.2.2. Comparison of the Samples Emotional Profile from the Original and the Adapted List

**Figure 5:**
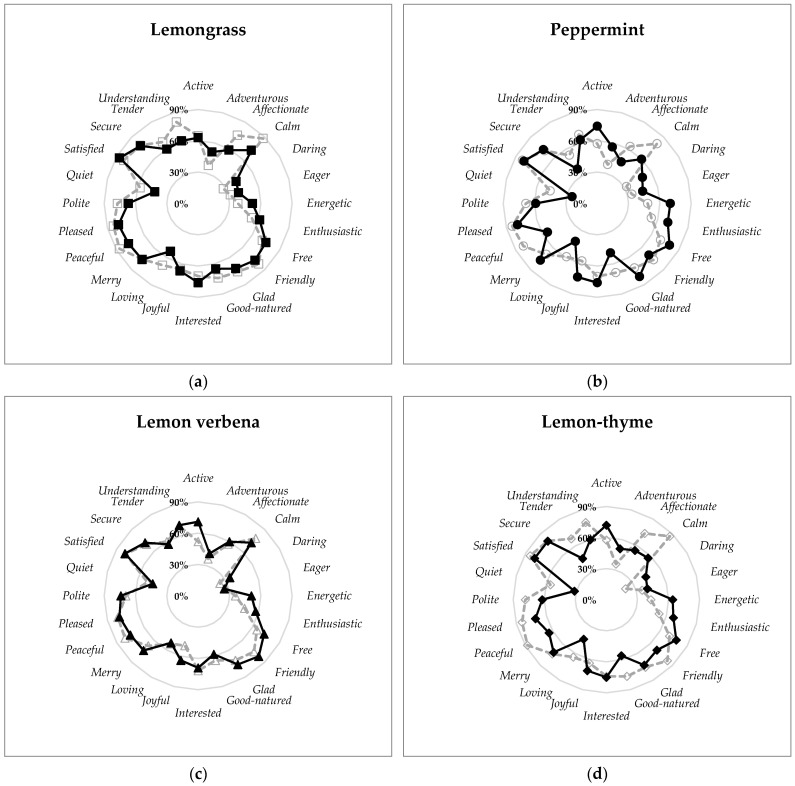
The emotional profile of the samples (**a**) lemongrass; (**b**) peppermint; (**c**) lemon verbena; and (**d**) lemon thyme from the evaluation with the original list terms (continuous line) and the one with the adapted list which intended to assess the impact of the harvesting conditions, of the average of treatments (dotted lines).

Figure 5 compares the emotional profile of the samples from the different treatments (type of cut × plant area) with the emotional profile of the correspondent sample from experiment I. Only the emotion terms common to both lists are presented. Generally, there is a similar characterization of the four treatment samples with low significant variances between them. When comparing with the equivalent sample from experiment I, one can observe that for lemongrass and lemon verbena the emotional profiles are similar, while for peppermint and lemon thyme there are some minor changes. For peppermint, some emotion terms such as *quiet*, *peaceful*, *calm*, *affectionate* and *good-natured* get a higher frequency of mention when the treatment samples are evaluated, while for other terms like *joyful*, *energetic* and *active* the frequency of mention is higher during the experiment I. For lemon thyme, similar behaviour is observed, *tender*,* quiet*, *pleased*,* peaceful*, *good-natured*, *calm* and *affectionate *were more elicited on the treatment samples’ evaluation, while *energetic* and *active* were more elicited on the experiment I evaluation. These differences can be explained due to the samples’ nature and also because the samples’ evaluation in both experiments was performed by different groups of consumers.

## 4. Discussion

One of the purposes of this research was to develop a shorter version of the EsSense Profile^®^, applied to a new food category—herbal infusions, following an emotional consumer lexicon adaptation. This approach has the advantage of balancing the cost of time and resources when compared to the pre-determined lexicons. For this, authors have combined the consumer voice into a specific product development process, benefiting from the emotional list available from literature, as consumers may not be able to articulate all their emotions during the experiment [8].

In fact, during Experiment I, authors found, through a consumer test (*n* = 100), that some of the emotion terms from the 39-emotion list of the EsSense Profile^®^ were not relatable, nor did they contribute to the discrimination of the herbal infusion products. Other studies by Bhumiratana et al. [51], Chaya, Eaton, Hewson, Vázquez, Fernández-Ruiz, Smart and Hort [13], Silva, Jager, van Bommel, van Zyl, Voss, Hogg, Pintado and de Graaf [14], Talavera and Sasse [52] reinforce the fact that the focus group methodology may be useful to define the final list of emotions. In fact, in this research, during the focus group sessions, participants mentioned that the EsSense Profile^®^ list was too extensive for herbal infusion products. Moreover, this approach allowed for the exclusion of irrelevant terms, thus shortening the list and removing potential consumer confusion [26] while maintaining relevant terms. Indeed, when the emotion terms were translated into Portuguese, some of them were perceived as synonyms (e.g., *tender* and *mild*), representing a certain redundancy upon consumer evaluation, thus leading to some degree of consumer fatigue. As a result, this approach allowed for an increasing discriminative ability of the lexicon [8], even considering that in this experiment consumers were not allowed to create from the beginning an emotional lexicon in their own words and discuss them [8,13].

Indeed, Jiang, King and Prinyawiwatkul [9], summarized some of the common criteria for emotion lexicon development that are in line with the decisions applied in the present research for the definition of the emotion lexicon for herbal infusions, such as, terms should: (a) be discriminating (exclusion of *nostalgic*, *wild* and *guilty*), (b) have high usage frequency (*aggressive*, *bored, disgusted* and *worried* removed since the frequency of usage was very low), (c) belong to the domain of emotion or to have no misunderstanding and no vague meaning (*pleasant* and *good* removed because they misled as sensory attributes) (d) not be redundant (for the Portuguese language *happy* is similar to *glad*; *steady* to *secure; mild* to *tame, warm* to *tender*).

The final consumer-validated list to evaluate herbal infusions contains 24 emotional terms, a shorter list with more distinct words. Despite some concerns that this new list may yield a lower performance, with terms that do not guarantee the differentiation of the products, just through the effect of having a reduction in the number of list terms, results have proven the ability of the shorter list to still discriminate between different herbal infusions. This customized list consists mostly of positive emotions, which is in agreement with the results obtained in other studies on product emotions, considering that food experiences are mainly positive [53,54]. This could also be explained by the fact that our panellists were all consumers of herbal infusions, with a tendency to have a positive emotional profile within this product category [16,55]. This means that the presence of regular consumers ensures the likelihood of a positive effect being evoked, because, as previously referred, consumers use positive emotions when describing foods [10], particularly for those that are more familiar with the product category [55,56]. Moreover, the hedonic evaluation of the herbal teas yielded very positive average liking, supporting that there was no clear need to include additional negative terms as in the work by Kuesten et al. [57], where the PANAS questionnaire was used to evaluate the emotional response to aromas of phytonutrient supplements.

The second purpose of this study was to evaluate the impact of the harvest conditions (the type of cut and plant part) on the evoked emotional profile. Despite the identification of no significant differences in the overall liking of the samples from the different harvest conditions, researchers found differences in the treatments related to the emotional profile of lemon verbena infusions, which later helped with the definition of the premium lot. This is in line with the works on food-evoked emotions by King and Meiselman [16] and Ng, Chaya and Hort [8], showing that the measurement of overall liking is not a sufficient benchmark to predict product success. The premium lot combination chosen was the Tips—Mechanical sample, which is the one with a more differentiated and intense emotional profile. Moreover, as presented by Rocha et al. [58], this premium lot was also significantly differentiated from other commercial samples of lemon verbena, particularly by its positive correlation with emotion-related terms *adventurous* and *energetic*.

## 5. Conclusions

The adapted version of the EsSense Profile^®^ presented a good potential to discriminate herbal infusions. The dynamic nature of the EsSense Profile^®^ emotions list was validated, meaning that it is not a static list of emotions, but one that can be adapted for the product category under evaluation. The importance of the consumers’ voice regarding the definition of the emotions list was emphasized, particularly regarding the meaning of the terms and the length of the list. Indeed, the emotional profiles evoked by the chosen herbal infusions gave an additional dimension to liking, in the sense that the herbal infusions evaluated in this research were equally liked, from a sensory point of view, but differed substantially in their emotional profile. It was shown that for different commercially available lemon verbena, yielding similar liking scores upon blind tasting, there were significant differences in the infusions-evoked emotional profile, with the premium lot being the one with stronger evoked emotions, such as *adventurous* and *energetic*. For this purpose, the consumers’ voice was combined with the EsSense Profile^®^, to adapt the last to the herbal infusions category. This adaptation into the specific product category was conveyed by gathering terms from consumers’ product-evoked emotions, elicited when they were thinking or experiencing the food product.

Small differences in outcomes of the evaluation of the type of cut and plant part can be justified by the organic production method or by the high quality of the plant used for the preparation of this infusion. On the other hand, the fact that people are forced to rationalize food-related emotions may condition their answers.

These results give an in-depth knowledge about the consumers’ emotional perception of herbal infusions. This information is useful for producers and markets, who may use this information to improve their communication strategies.

## Figures and Tables

**Table 1:** Temperature and time of hot water for infusions preparation.

Sample	Temperature (°C)	Time (Minutes)
Lemon balm (*Melissa officinalis*) ^1^	85	5.0
Chamomile (*Chamomilla recutita*) ^1^	90	5.0
Globe amaranth (*Gomphrena globose*) ^1^	90	10.0
Tutsan (*Hypericum androsaemum*) ^1^	85	7.0
Lemongrass (*Cymbopogon citratus*) ^2^	99	8.5
Lemon verbena (*Aloysia triphylla*) ^3^	96	6.0
Peppermint (*Mentha x piperita*) ^2^	95	4.0
Lemon thyme (*Thymus x citriodorus*) ^2^	92	7.0

^1^ According to packaging/producer instructions; ^2^ from Rocha [37]; ^3^ from Rocha [38].

**Table 2:** Mean overall liking scores for the different herbal infusions under evaluation during the development of the emotional profile ballot.

Infusion	Overall Liking ^1^ Mean (±SD)
Peppermint (*Mentha x piperita*)	7.9 (±1.3) ^a^
Lemongrass (*Cymbopogon citratus*)	7.8 (±1.3) ^a^
Lemon verbena (*Aloysia triphylla*)	7.7 (±1.5) ^a^
Lemon thyme (*Thymus x citriodorus*)	7.5 (±1.7) ^a^
Lemon balm (*Melissa officinalis*)	7.5 (±1.4) ^a^
Globe amaranth (*Gomphrena globose*)	7.0 (±1.3) ^b^
Chamomile (*Chamomilla recutita*)	6.9 (±1.7) ^b^
Tutsan (*Hypericum androsaemum*)	6.4 (±1.8) ^c^

^1.^ Overall liking ranging between: 1—Dislike extremely and 9—Like extremely; ^a,b,c^ homogeneous groups according to the Wilcoxon non-parametric test, at a 95% confidence level.

**Table 3:** List of emotion terms used in both experiments. In parenthesis, the Portuguese version as it was used for both experiments (from Cunha [50]); the (a) at the end of some terms represents the masculine/feminine gender-specific variation (e.g., *Ativo(a)* reads as *Ativo*/*Ativa*).

Kept Emotion Terms—EN (*PT*)		Removed Emotion Terms—EN (*PT*)
Active (*Ativo(a)*)	Interested (*Interessado(a)*)	Aggressive (*Agressivo(a)*)
Adventurous (*Aventureiro(a)*)	Joyful (*Jovial*)	Bored (*Aborrecido(a)*)
Affectionate (*Carinhoso(a)*)	Loving (*Amoroso(a)*)	Disgusted (*Enojado(a)*)
Calm (*Calmo(a)*)	Merry (*Alegre*)	Good (*Bem*)
Daring (*Ousado(a)*)	Peaceful (*Em paz*)	Guilty (*Culpado(a)*)
Eager (*Ávido(a)*)	Pleased (*Agradado(a)*)	Happy (*Feliz*)
Energetic (*Energético(a)*)	Polite (*Amável*)	Mild (*Meigo(a)*)
Enthusiastic (*Entusiasmado(a)*)	Quiet (*Quieto(a)*)	Nostalgic (*Nostálgico(a)*)
Free (*Livre*)	Satisfied (*Satisfeito(a)*)	Pleasant (*Agradável*)
Friendly (*Amigável*)	Secure (*Seguro(a)*)	Steady (*Firme*)
Glad (*Contente*)	Tender (*Terno(a)*)	Tame (*Dócil*)
Good-natured (*Bondoso(a)*)	Understanding (*Compreensivo(a)*)	Warm (*Caloroso(a)*)
		Whole (*Completo(a)*)
		Wild (*Descontrolado(a)*)
		Worried (*Preocupado(a)*)

**Table 4:** Overall liking of the herbal infusions used during experiment II and for each treatment evaluated (plant part and type of cut).

Herbal Infusions	Treatment	Treatment Overall Liking ^1^ Mean ^a^ (±SD)	Infusion Overall Liking ^1^ Mean (±SD)
Lemongrass	2nd Half—Manual	7.6 (±1.0)	7.7 (±0.9)
	2nd Half—Mechanical	7.8 (±0.8)	
	Tips—Manual	7.8 (±0.9)	
	Tips—Mechanical	7.7 (±1.0)	
Peppermint	2nd Half—Manual	7.5 (±1.4)	7.6 (±1.2)
	2nd Half—Mechanical	7.6 (±1.2)	
	Tips—Manual	7.7 (±1.3)	
	Tips—Mechanical	7.6 (±1.0)	
Lemon verbena	2nd Half—Manual	7.8 (±1.0)	7.7 (±1.0)
	2nd Half—Mechanical	7.6 (±1.2)	
	Tips—Manual	7.7 (±0.9)	
	Tips—Mechanical	7.6 (±1.1)	
Lemon thyme	2nd Half—Manual	7.5 (±0.9)	7.5 (±0.9)
	2nd Half—Mechanical	7.5 (±0.9)	
	Tips—Manual	7.6 (±0.8)	
	Tips—Mechanical	7.4 (±1.0)	

^1.^ Overall liking ranging between: 1—Dislike extremely and 9—Like extremely; ^a^ With no significant difference between treatments, for each herbal infusion, according to the Friedman test, at a 95% confidence level.

## Data Availability

The data presented in this study are available on request from the corresponding author, following request from the company involved in the projects.
